# Nanosensitive optical coherence tomography for detecting structural changes in stem cells

**DOI:** 10.1364/BOE.485082

**Published:** 2023-03-03

**Authors:** Anand Arangath, Niamh Duffy, Sergey Alexandrov, Soorya James, Kai Neuhaus, Mary Murphy, Martin Leahy

**Affiliations:** 1 Tissue Optics and Microcirculation Imaging Facility, Physics, School of Natural Sciences, University of Galway, Galway, Ireland; 2 Regenerative Medicine Institute, University of Galway, Galway, Ireland; 3 Casey Eye Institute, Oregon Health & Science University, Portland, OR 97239, USA; 4 The Institute of Photonic Sciences (ICFO), Barcelona, Spain

## Abstract

Mesenchymal stromal cells (MSCs) are adult stem cells that have been widely investigated for their potential to regenerate damaged and diseased tissues. Multiple pre-clinical studies and clinical trials have demonstrated a therapeutic response following treatment with MSCs for various pathologies, including cardiovascular, neurological and orthopaedic diseases. The ability to functionally track cells following administration in vivo is pivotal to further elucidating the mechanism of action and safety profile of these cells. Effective monitoring of MSCs and MSC-derived microvesicles requires an imaging modality capable of providing both quantitative and qualitative readouts. Nanosensitive optical coherence tomography (nsOCT) is a recently developed technique that detects nanoscale structural changes within samples. In this study, we demonstrate for the first time, the capability of nsOCT to image MSC pellets following labelling with different concentrations of dual plasmonic gold nanostars. We show that the mean spatial period of MSC pellets increases following the labelling with increasing concentrations of nanostars. Additionally, with the help of extra time points and a more comprehensive analysis, we further improved the understanding of the MSC pellet chondrogenesis model. Despite the limited penetration depth (similar to conventional OCT), the nsOCT is highly sensitive in detecting structural alterations at the nanoscale, which may provide crucial functional information about cell therapies and their modes of action.

## 1. Introduction

Regenerative medicine deals with the use of stem/progenitor cells to repair, replace or regenerate damaged tissues or organs. Therapeutic approaches in regenerative medicine are pitched towards the inherent self-regenerating ability of tissues and cells, primarily stem cells. Mesenchymal stromal cells (MSCs) have been at the forefront of regenerative medicine and tissue engineering since the late 1960s due to their capacity to differentiate into multiple cell types, including cells of the musculoskeletal system such as chondrocytes, adipocytes and osteoblasts [1,2]. MSCs are a population of multipotent stromal cells which can be isolated from adult and foetal tissues like bone marrow, placenta and umbilical cord [3–5]. Traditionally, the therapeutic mechanism of action of MSCs was proposed to occur due to local engraftment following administration and direct differentiation to replace damaged/diseased cells [6]. This theory of MSC engraftment at the site of injury is still plausible, but the predominant factor governing their therapeutic potency is now thought to be primarily attributed to their immunomodulatory properties [7–9]. MSCs have been shown to regulate multiple types of immune cells in inflammatory-rich cytokine environments through various mechanisms. These include macrophages [10] via the promotion of an anti-inflammatory M2 phenotype (macrophage polarisation), and T-cell suppression [11] via indoleamine 2,3-dioxygenase (IDO) release, as well as inhibition of dendritic cell maturation by inhibiting and increasing the secretion of TNF- α and IL-10, respectively [12]. Pre-clinical studies using MSCs have demonstrated efficacy, but the clinical landscape has presented low-quality evidence due to a lack of standardisation of cell preparation and study design and an incomplete understanding of the homing, engraftment and biodistribution of cells transplanted in vivo.

In order to enhance the translation of these therapies to the clinic, biomedical imaging and tracking cells following administration in vivo are critical to understanding their homing, engraftment, biodistribution and differentiation. Currently, many imaging modalities are available for use in stem cell tracking applications [13,14]. The ideal imaging modality requires high sensitivity, high spatial resolution, high temporal resolution, the ability to image for longer durations and non-toxic imaging. Magnetic resonance imaging (MRI) can provide long-term tracking and high spatial resolution. However, its poor temporal resolution limits the real-time imaging capability, and low sensitivity demands high doses of contrast agents. High doses of iron oxide contrast agents could impact the therapeutic capacity and potency of MSCs as it reduces their differentiation potential. [15–17]. Computed tomography has good temporal resolution but utilises ionising radiations and has poor sensitivity [18,19]. Radioactive imaging techniques like positron emission tomography and single-photon emission computed tomography have low spatial resolution and are not suitable for long-term imaging due to the short half-lives of the radioisotopes [15,16]. Optical methods like fluorescence and bio luminescence imaging have high resolution and sensitivity. However, the shallow penetration depth and phototoxicity of the imaging dyes limit their scope for clinical applications [15,16,20,21]. Photoacoustic imaging (PAI) methods are steadily evolving for pre-clinical and clinical applications in stem cell therapy. They have high resolution, high penetration depths, and facilitate real-time imaging and prolonged observation periods. PAI cannot visualise stem cells directly as the cells do not have an adequate optical absorption coefficient. However, stem cells can be monitored using PAI after labelling them with suitable exogenous contrast agents [22–24]. Recently, dual plasmonic gold nanostars with absorption maxima at 1064 nm were developed, which is suitable for use as a contrast medium for PAI. These nanoparticles generate a robust photo-thermal signal in response to light of longer wavelengths [25]. Combining the novel nanoparticles with PAI has the potential to offer a class-leading imaging solution for stem cell therapies. However, to accurately determine the different stages of stem cell differentiation and therapeutic activation at the cellular level, imaging techniques that can visualise the internal submicron structure in real-time with nanoscale sensitivity are desired.

Optical coherence tomography (OCT) is a non-invasive imaging technique based on low-coherence interferometry [26]. Conventional OCT promises depth-resolved, label-free, real-time, 3-dimensional imaging of tissues and materials with a spatial resolution in a few micrometres. In contrast to optical depth-resolved imaging techniques like confocal microscopy and two-photon microscopy, OCT has a much better speed and a higher imaging range of about 2 mm. However, the spatial resolution of most OCT systems is not sufficient for cell imaging. Modern OCT systems that can image cellular and subcellular structures [27–29] are expensive, difficult to operate and require the sample to be sufficiently stabilized. Therefore, the use of OCT systems for stem cell research is either based on photothermal responses to extrinsic contrast agents such as nanoparticles or in combination with other imaging techniques [13]. Functional imaging techniques in OCT such as phase-sensitive OCT, speckle OCT and inverse spectroscopic OCT can detect structural changes smaller than the size of a coherent gate [30–32]. Recent research by Alexandrov et al. has discovered a label-free process for detecting changes at the nanometre scale using nanosensitive optical coherence tomography (nsOCT) [33]. Nanosensitive OCT is a technique based on the spectral encoding of spatial frequency, which can significantly improve the sensitivity to structural alterations and spatial resolution, enabling the possibility to provide important additional information about morphological changes at the nanoscale level. The nsOCT method allows conventional OCT systems to be extended for performing indirect cellular and subcellular structural analysis and monitoring without requiring expensive optical components and engineering. [33–41].

The potential of nsOCT for assessing wound healing in the cornea, monitoring tumour growth, and structural changes in the cornea associated with cross-linking treatment is already reported in the literature [36,37,41]. Nanosensitive OCT, providing structural information with nanoscale sensitivity and high temporal resolution, can be used for long-term tracking, and is label-free. However, the morphological information provided by nsOCT will be unspecific, and similar to conventional OCT, as the penetration depth is limited to less than a millimetre in most cases. It has to be kept in mind that no imaging strategy will address all issues and multimodal imaging systems are required to exploit one system’s advantages to compensate for the disadvantages of another. Therefore, to demonstrate nsOCT’s potential to provide structural information relating to stem cells at the nanoscale level, we have designed two experiments: 1. nsOCT detection of varying concentrations of nanostars following internalization by cells and 2. nsOCT detection of MSC chondrogenic differentiation. For the first experiment, the nanostars used to label MSCs were originally developed as a contrast agent for stem cell tracking in conjunction with PAI, as previously mentioned. The labelling processes involve directly adding the nanoparticles to the cell culture medium, which are endocytosed by the cells and packaged into intracellular compartments. Because MSCs are primary cells, they are a heterogeneous population that during in vitro expansion may be present at different stages of the cell cycle. As a result, nanoparticles are not internalized by all cells unanimously or at the same time/rate, leading to different quantities of nanoparticles in different cells. Furthermore, in vivo administration of cells may initiate processes such as apoptosis, migration, proliferation and differentiation. Such processes may dilute the signal at the engraftment site due to potential exocytosis from the cells following apoptosis or dilution of the label to daughter cells following proliferation/division. Therefore, it is essential to consider how we can monitor different numbers of stem cells with different concentrations of nanostars while investigating the regenerative pathway in vivo. The nanoscale sensitivity of nsOCT can provide quantitative structural information related to different concentrations of nanostars inside cells. Our results suggest that the dominant spatial period of the MSCs in pellet culture increases with increasing concentrations of nanostars. We correlate our results from nsOCT analysis with Fourier analysis on images acquired using a transmission electron microscope (TEM).

With respect to the second experiment, nsOCT has been utilised to monitor stem cell differentiation in vitro. This work has been previously published examining the direct comparison of positive (chondrogenic differentiation) and negative (undifferentiated controls) cells [39]. In the present study, this work has been further elaborated to provide information on additional time points of differentiation and how the structural composition of the samples changes throughout this process. The previous publication [39] compared two samples: positively differentiated cells (chondrogenic pellets) and undifferentiated controls. The results of this analysis indicated a lower mean spatial period for differentiated cells in comparison to their undifferentiated counterparts. Our current study adds to this experiment by performing nsOCT imaging at multiple stages of the differentiation process, and found the same result, demonstrating the reproducibility of the system for this indication. The current study also provides additional information about the trend in structural changes that occur during cartilage development over time, indicating a slight increase in spatial periods during the early condensation phases followed by a gradual decline during the transition to a proteoglycan and collagen type II-rich matrix. These in vitro findings have provided a significant foundation for understanding how the structure change with increasing cartilage matrix formation. By contrast, a gradual decline and breakdown of the cartilage occur over time during OA pathogenesis; this decline occurs at a very slow rate. The current study demonstrates that nsOCT can detect subtle changes in cartilage formation during in vitro MSC chondrogenesis and may therefore be capable of detecting subtle changes in cartilage degradation in in vivo OA settings. This work is not only relevant to MSCs as a therapeutic but is a useful model for assessing the presence or absence of cartilage matrix within a sample. This analysis is relevant to a condition known as osteoarthritis (OA). OA is a chronic, degenerative joint disorder and the most common cause of disability worldwide. It is characterised by subchondral bone remodelling, synovitis, meniscal damage and osteophyte development, but the major hallmark of OA is the degradation and loss of the articular cartilage [42]. One of the most prominent factors in OA pathogenesis is a dysregulation of cytokine balance favouring pro-inflammatory cytokines such as IL-1 β , TNF- α and IL-6 [43]. These cytokines mediate multiple signalling pathways that activate other cytokines and induce inflammatory cell infiltration, further releasing pro-inflammatory factors and disease progression. These factors shift chondrocyte homeostasis towards a catabolic phenotype by inducing the production of matrix-degrading enzymes such as aggrecanases (A Disintegrin and Metalloproteinase with Thrombospondin Motifs 5 (ADAMTS-5)) and collagenases (matrix metalloproteinase 13 (MMP-13)) [44]. This results in proteoglycan loss and degradation of the collagen network in cartilage. Early diagnosis of OA is critical to ensure effective treatment strategies are implemented before the severe disease is established. However, the development and establishment of OA is known to occur long before symptomatic disease presents. Changes in the composition of the articular cartilage during disease onset occur at the molecular (nanoscale level) [45]. Following a dysregulation in chondrocyte homeostasis, the collagen network begins to degrade, resulting in proteoglycan loss and breakdown of the extracellular matrix (ECM) surrounding those cells. Currently, none of the imaging modalities used in OA diagnostics can effectively detect the onset of these changes. nsOCT is capable of providing nanometre-structural information about a sample and may have the potential to detect such changes and be useful in diagnosing early OA. By exploiting the chondrogenic potential of MSCs, we have validated nsOCT to distinguish between a sample with a high proteoglycan and ECM (differentiated sample) content and one that does not contain any cartilage-like proteins (undifferentiated sample). Our results have shown that nsOCT can provide information related to the structural changes accompanying different stages of chondrogenic differentiation of MSCs in pellet culture, and we have correlated the results with histological analysis. The preliminary results presented in this manuscript are encouraging. They suggest that the nsOCT technique can be used in parallel with other imaging modalities like PAI to obtain additional information relevant to tracking the therapeutic effects of stem cells based on associated morphological changes.

## 2. Theory and methods

### 2.1 Experimental design

Four experimental groups were used in this study. The first three groups aim to evaluate the capacity of nsOCT to distinguish MSCs labelled with different concentrations of nanostar. We prepared the first and the second experimental groups to test the reproducibility of the results under different conditions. The first group consisted of nine MSC pellets from the same donor. Among these samples, there are three replicates, each of unlabelled cells, cells labelled with 100 pM concentration of nanostars and cells labelled with 400 pM concentration of nanostars, all in pellet culture. The second group of samples consisted of three samples of MSCs from the same donor encapsulated in sodium alginate. The three samples are MSCs without labelling, labelled with 100 pM and 400 pM concentrations of nanostars. The third experimental group comprises MSCs in pellet culture from three different donors. We prepared five MSC pellets from each donor, one control and four other pellets labelled with different concentrations (100 pM, 200 pM, 300 pM and 400 pM) of nanostars. The fourth experimental group was designed to study the potential of nsOCT to detect the morphological changes associated with different stages of chondrogenic differentiation of stem cells. In this group, we prepared MSCs from three different donors in pellet culture. From each donor, we produced five samples representing different stages of chondrogenic differentiation ( control, day 1, day 4, day 7 and day 21). To correlate with the nsOCT results from experiment groups 1 and 4, we did corresponding transmission electron microscopy and histology imaging, respectively.

### 2.2 Gold nanostars

Gold nanostars, composed of a spherical core and multiple branches, were chosen for this study. These nanostars were originally developed as a contrast agent for photoacoustic imaging. They can localise the electron density at their sharp tips, thus maximising their effective cross-sectional area for interaction with photons [25,46]. Moreover, anisotropic nanoparticles like gold nanostars have unique optical properties for biological imaging in the second near-infrared window. While labelling stem cells with contrast agents, it is crucial to ensure that their viability, differentiation, migration and therapeutic potential are not compromised. Biocompatibility and colloidal stability can be achieved by PEGylating nanoparticles with neutral/ hydrophilic polymers. For tracking stem cells longitudinally, high cellular uptake and retention are preferred. In our study, we are using gold nanostars which were synthesised using the seed-mediated growth method at room temperature, followed by the addition of HS-PEG-COOH solution for PEGylation of the gold nanostars with carboxyl groups. The carboxylated gold nanostars were then centrifuged and dispersed in a chitosan oligosaccharide lactate solution allowing the formation of a uniform layer of Chitosan coating, thus making them favourable for long-term stem cell monitoring in vivo [47,48]. The nanostars have a hydrodynamic size of 92 nm. The hydrodynamic measurement was carried out using the Anton Paar Litesizer 500, which employs the principle of dynamic light scattering using three detection angles (side, back, or forward scattering). The angle for light scattering was selected automatically by the instrument, and 60 runs were carried out for the measurement.

### 2.3 Preparation of MSC pellets labelled with different concentrations of nanostars

Human MSCs were isolated from bone marrow following bone marrow aspiration from the iliac crest of healthy donors. The cells were isolated based on plastic adherence in culture and expanded in alpha-Minimum Essential Medium supplemented with 10% foetal bovine serum, 1% penicillin/ streptomycin, and 1 ng/ml recombinant human basic fibroblast growth factor [49]. All cells were characterized before use according to the International Society for Cell and Gene Therapy (ISCT) [50]. Flow cytometric analysis was performed to confirm MSCs were positive for CD105, CD73 and CD90 and negative for CD3, CD34, CD14, CD45, CD19 and HLA-DR surface antigen expression, as well as capable of differentiation to the adipogenic, osteogenic and chondrogenic lineages using previously described methods. [51] Cells were expanded until passage 3 and labelled with either 100 pM, 200 pM, 300 pM or 400 pM of nanostars for 24 hours. Unlabelled controls were also prepared and maintained in culture for 24 hours. Cells were washed to remove residual nanostars, harvested and counted. Cell pellets were then prepared for each of the three conditions, each pellet containing 250,000 cells. The pellets were maintained in a chondrogenic medium for 7 days. This was not to induce complete differentiation but to ensure a condensation step was initiated to maintain a 3-dimensional structure that would not dissociate during preparation for imaging. Three technical replicates were prepared for each sample to assess potential variability between pellets.

### 2.4 Encapsulation of MSCs in sodium alginate

For assessment of nsOCT imaging to detect nanostars in MSCs within a biomaterial gel, MSCs were first labelled overnight for 24 hours with either 100 or 400 pM nanostars. Unlabelled MSCs without nanostars were included as a control. MSCs were encapsulated in sodium alginate using a previously described method. [52]. In brief, A 1.2% sodium alginate gel was prepared by dissolving UV sterilized alginic acid sodium salt in 0.15M sodium chloride (NaCl). This solution was then sterile filtered through a 0.22 μ M filter. Labelled cells were then trypsinised, counted and centrifuged at 300 g for 5 minutes. The cell pellet was resuspended in 1.2% sodium alginate solution at a density of $8\times 10^{6}$ cells/ml. Alginate beads encapsulating the cells were then produced by pipetting 100 μ l of alginate-cell suspension in a 1000 μ l pipette tip and allowing 3-4 droplets to fall from a distance of 15 cm into 102 mM calcium chloride (CaCl2) to allow sodium alginate crosslinking. 3-4 beads per sample yielded approximately $2.5\times 10^{5}$ cells/bead. After 10 minutes in $CaCl_{2}$ , the beads were washed 3 times with 0.15 M NaCl for 10 minutes each. The beads were then cultured for 7 days in a human MSC medium (MEM- α , 10% foetal bovine serum, 1% penicillin/streptomycin and 5 ng/ml fibroblast growth factor-2). After 7 days, the beads were washed 3 times in D-PBS and fixed with 10% neutral-buffered formalin supplemented with 102 mM $CaCl_{2}$ for 1 hour. Following fixation, the samples were washed 3 times in D-PBS and nsOCT imaging was performed.

### 2.5 TEM imaging

Transmission electron microscopy was performed to compare the uptake and intracellular localisation of various concentrations of nanostars. MSCs were incubated overnight for 24 hours with 100, 200, 300 or 400 pM nanostars. Unlabelled MSCs without incubation of nanostars were used as a control. Following incubation, the cells were washed 3 times in D-PBS and fixed as a monolayer using 2% glutaraldehyde + 2% paraformaldehyde in 0.1 M sodium cacodylate buffer pH 7.2 for 2 hours. Following fixation, cells were gently scraped into the solution and centrifuged at 300 g for 5 minutes. Samples were then re-suspended and post-fixed in a secondary fixative solution (1% osmium tetroxide in 0.1 M sodium cacodylate buffer pH 7.2). The cells were then dehydrated through a graded series of ethanol (30%, 50%, 70%, 90% and 100% for 2×15 minutes each), acetone for 2×20 minutes followed by a graded series of resin and acetone (50:50 resin: acetone for 4 hours, 75:25 resin: acetone overnight, 100% resin for 6 hours). Finally, the cells were placed in 100% resin for 48 hours at 65°C for polymerisation. Ultrathin sections (70 nm) were then cut from the resin blocks using a diamond knife and mounted on 3 mm copper grids. The sections were then stained with uranyl acetate and lead citrate to enhance contrast and allowed to air dry. Representative images for each sample were taken at 50,000x magnification using a Hitachi H7500 transmission electron microscope. Fourier analysis was then performed

### 2.6 Preparation of MSC pellets to evaluate different stages of chondrogenic differentiation

The MSCs expanded to passage 3 were induced to undergo chondrogenic differentiation using micromass 3-D pellet culture at 2% $O_{2}$ and 10 ng/ml transforming growth factor (TGF)- β 3 [53]. All cell pellets contained $2.5\times 10^{5}$ [53] cells and were maintained in culture for either one, four, seven or twenty-one days, followed by fixation in 10% neutral buffered formalin. Pellets used for nsOCT imaging were dehydrated through an alcohol gradient (70%, 90%, and 100% for 1 hour each) to prevent changes in the hydration states of pellets interfering with imaging. For all the procedures performed using human cells, we received consent from donors and approval from the Clinical Research Ethical Committee at University College Hospital, Galway, Ireland.

### 2.7 Histology

MSC pellets treated to undergo chondrogenic differentiation were automatically processed using a tissue processor. The processing included cycles of 70% IMS for one hour, 90% IMS for one hour, 100% IMS for three hours, xylene for three hours and melted paraffin wax for three hours. The pellets were then embedded in paraffin wax, sectioned into 5 μ m sections and mounted onto slides for staining. The pellets were stained with 0.1% Safranin-O and 0.1% fast-green and imaged using an Olympus 1X71 microscope. An objective lens with a numerical aperture of 0.40 was used.

### 2.8 OCT imaging

For the OCT imaging, a commercial spectral domain system, Telesto 3, from Thorlabs, Inc., New Jersey, United States was used. The system has a spectral bandwidth from 1180 nm up to 1415 nm, centred at 1295 nm. The spectrometer features a 2048 pixel CCD detector. The specifications allow an axial resolution of 5.5 μ m and imaging depth of 3.6 mm in air. The LSM03 objective lens was used for all the imaging in this study. The objective lens has a numerical aperture of 0.055 and a lateral resolution of 13 μ m. The image was acquired at a rate of 76 kHz with a promised sensitivity of 96 dB. For experimental groups 1, 2 and 3, 3-D volumes were acquired with 500 pixels each in the lateral directions (0.5 mm x 0.5 mm). For experimental group 4, we acquired images with 300 pixels in each lateral direction (0.3 mm x 0.3 mm).

### 2.9 Nanosensitive optical coherence tomography

**Fig. 1.**
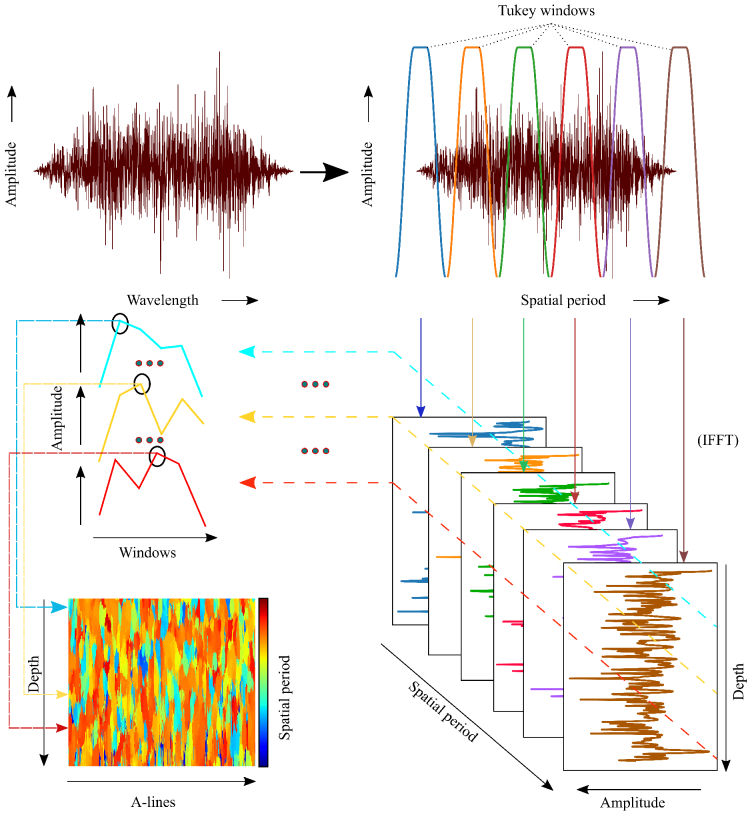
A flow chart of nsOCT’s image formation. The spatial period values replace the wavelength axis of the interference spectrum. Then the spectrum is decomposed to a fixed number of sub-bands using Tukey windows. In this flow-chart, we show six sub-bands. Within the limited bandwidth of each sub-band, FFT is performed to form depth profiles or A-lines. Therefore, in this case, for each voxel, energy contributions from 6 sub-bands will be present. Then we form an energy contribution versus the corresponding spatial period of the sub-band plot, called the spatial period profile. From this spatial period profile, we determine the spatial period value corresponding to the highest energy. The spatial period value thus determined is colour-coded to represent the respective voxel [37,41].

As described by the general scattering theory, information about the high spatial frequencies of the object is present in the OCT signal. A detailed theoretical analysis of the scattered field from an object in reflection configuration is presented in Alexandrov et al. [39]. This spectrally encoded high spatial frequency information present in the OCT signal is sensitive to structural features and hence can be used to detect structural changes within the sample. In an OCT signal, where the scattering and illumination angles are close to zero, the collected axial spatial frequency can be expressed as [39]

(1) $$\nu_{z}=\frac{2n}{\lambda }$$

where n is the refractive index, and λ is the wavelength. From this equation, we can see a one-to-one correspondence between the wavelength and axial spatial frequency. Therefore, the spectral interference signal in Fourier-domain OCT has one-to-one correspondence between the complex amplitude of axial spatial frequency components and wavelengths. The bandwidth of the source ( δλ ) determines the range of spatial frequencies ( δν ) that can be detected using OCT by the relation [39]

(2) $$\delta \nu =\frac{2n\delta \lambda }{\lambda_{1}\cdot \lambda_{2}}$$

where $\lambda_{1}$ and $\lambda_{2}$ are the shortest and longest wavelengths of the source. However, during the Fourier transform in conventional OCT signal processing, the information regarding this high spatial frequency content is lost. Nanosensitive OCT is a method to translate this spatial frequency information from the Fourier domain to the image domain as wavelengths. In this manuscript, we only discuss different steps required to form the nsOCT images from the spectral interferogram. The pre-processing steps like the DC removal and K-space linearization apply to both nsOCT and conventional OCT signal processing. For nsOCT image formation, after the pre-processing steps, the spectral interference signal is re-scaled into spatial frequencies. The re-scaling is achieved by replacing the wavelength axis with the spatial period values available to capture as per Eq. (2). The spatial period is the reciprocal of spatial frequency. The objective is to determine the energy contribution from available spatial periods (structural sizes) in the overall interference spectrum. To achieve this goal, after re-scaling, we decompose the interference spectrum into a fixed number of sub-bands using the same number of equally spaced Tukey windows (Fig. 1). The number of windows can be selected for each application to provide the best relation between spatial and spectral resolution of nsOCT. The number of points in axial spatial frequency profiles will be equal to the number of windows selected. [39]. The Tukey windows are zero-padded to match the entire length of the array. Each Tukey window represents a particular spatial period determined by its position in the spatial period axis. We apply Fast Fourier Transform (FFT) to the spectrum within each sub-band to get the depth profile (A-scan). Therefore, at each depth, we will have information regarding the energy contribution (absolute of FFT) from all the sub-bands. Using this information, we construct spatial period profiles (Fig. 1) at each depth position (each pixel or voxel). It is possible to reconstruct the axial spatial frequency/period profiles at each small volume of interest within the 3D image. Then the nsOCT image can be formed as a colour map of some informative parameters of these profiles, including correlation between profiles, etc. To form the nsOCT images in this study, we find the spatial period value with maximum energy contribution in the spatial period profile. Each voxel is then colour-coded with this maximum spatial period to form the nsOCT image. Experimental validation of the nsOCT technique using phantoms with known sub-micron structures is available in the literature [37,39,41]. A flowchart describing the processing steps involved in nsOCT image formation is presented in Fig. 1.

## 3. Results and discussions

**Fig. 2.**
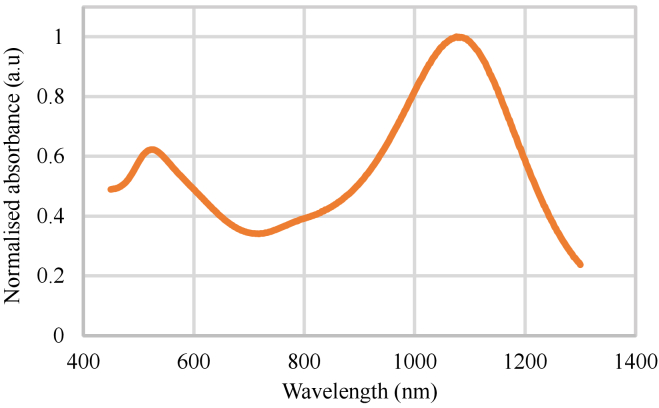
The absorption spectrum of the nanostars used to label the stem cells in this study. The nanostars have an absorption peak of 1076 nm and a hydrodynamic size of 92 nm. The spectral bandwidth of the OCT system used was between 1180 nm and 1415 nm.

The resonance wavelength for nanostars used in this study was out of the detected spectral bandwidth. The absorption spectrum of the nanostars is shown in Fig. 2. The UV-Vis-NIR absorption measurements were performed with a Shimadzu UV-2600 (Japan), and the samples were measured in polystyrene cuvettes (BrandTech, Fischer Scientific Ireland). The nanostars have an absorption peak at 1076 nm and a hydrodynamic size of 92 nm. The spectrum of the OCT system used ranged from 1180 nm and 1415 nm. Therefore, the signal from nanostars was weak. If the absorption peak of the nanostars is within the spectral bandwidth of the OCT system, it modifies the OCT signal, and such changes presumably could be detected even by conventional OCT [54–56]. In our case, when the absorption peak is outside the OCT bandwidth, we do not have any changes (or very weak changes) in the OCT signal directly from the nanostars. However, the nsOCT detects structural deformations induced by the introduction of nanostars. The scattering properties are directly affected by nanostars internalised and packaged by cells into multiple endocytic vesicles. As shown by TEM (Fig. 5), the internalisation rate by MSCs of these chitosan nanostars is very high, resulting in nanostars compartmentalised into numerous endocytic vesicles inside the cell. Studies suggest that the complexity of the cells has increased after internalisation of the nanostars [57]. Consequently, the different concentrations of nanostars could modify the nanoscale structure, which can be detected by nsOCT, even if the absorption is not within the wavelength range of the illumination source. Since nsOCT detects the dominant periodicity of the structure followed by nanoparticle internalisation, it could provide additional sensitivity to concentration. We applied nsOCT to find the spatial period changes among MSC pellets with different nanostar concentrations. The changes detected are small but still significant.

**Fig. 3.**
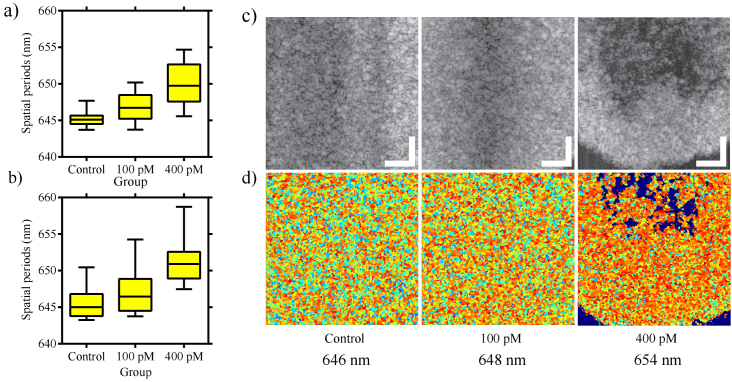
a) En face mean axial spatial periods from experimental group 1, represented in the box plot. Experimental group 1 consisted of three samples for control, 100 pM nanostar concentration and 400 pM nanostar concentration. It was observed that the spatial period values tend to be higher for higher concentrations of nanostars. b) represents the en face mean spatial periods from experimental group 2. Experimental group 2 consists of unlabelled MSCs, MSCs labelled with 100 pM concentration of nanostars and MSCs labelled with 400 pM concentration of nanostars, cultured in a sodium alginate matrix. The spatial period values tend to be higher for higher concentrations of nanostars. c) shows the representative en face images after conventional intensity-based OCT processing. The images were acquired from experimental group 1. All the images were recorded from the same depth. d) represents the nsOCT images corresponding to the images on (c). Below the images, the mean spatial period value from the respective nsOCT en face images is mentioned. The scale bar represents 100 µm.

We used three experimental groups (section 2.1) to evaluate the capacity of the nsOCT technique to detect MSCs labelled with different concentrations of nanostars. Experimental groups 1 and 2 were used to assess the potential variability between the pellets and reproducibility in different cell cultures, respectively. To form the box plots, we acquired 3-dimensional OCT images from each sample with 500 pixels in each lateral direction. From these 3-D volumes, we formed en face images at every slice along the depth. From these en face images, we then calculated the mean spatial period corresponding to that depth. The mean spatial period values from all the depths were assembled to constitute the box plot for each sample. Please note that for all the box plots presented in this manuscript, the horizontal line within the yellow box represents the median value in the box plots. The horizontal lines above and below the median value represent the upper and lower quartiles, respectively. The small horizontal lines above and below the box represent the upper and lower extremities, respectively. Each vertical solid line connecting either the upper or lower quartiles to the upper or lower extreme values represents 25% of the data points. Similarly, the rectangles above and below the median value account for 25% of the data points. The results from experimental group 1, which consisted of three samples each for control, 100 pM nanostar concentration and 400 pM nanostar concentration, are presented in Fig. 3(a). In Fig. 3(a), the box plot for each group consists of mean spatial periods from three duplicate samples from the same donor. Figure 3(b) shows results from the nsOCT analysis on experimental group 2. Experimental group 2 consists of unlabelled MSCs, MSCs labelled with 100 pM concentration of nanostars and MSCs labelled with 400 pM concentration of nanostars, cultured in a sodium alginate matrix. We used only one sample for each group. Figures 3(c) and 3(d) show representative conventional OCT and nsOCT images, respectively from experimental group 1. The darker shades in the OCT images are due to weak signal. From the nsOCT images, we can observe that the higher spatial periods become more dominant as the nanostar concentration increases. The mean spatial periods calculated from the en face images are mentioned below respective images.

**Fig. 4.**
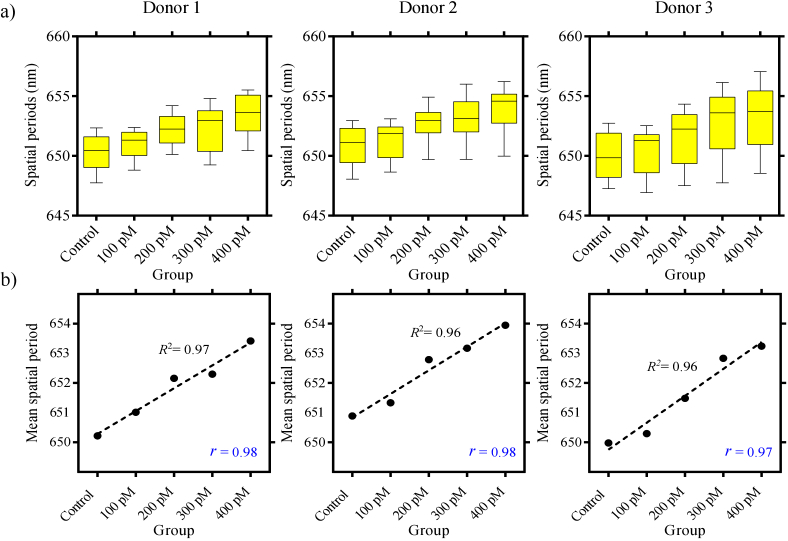
a) Three sets of box plots corresponding to three different donors (experimental group 3) are shown. The box plots are formed using mean spatial periods calculated from en face images. For each donor, there are five box plots corresponding to samples labelled with varying concentrations (unlabelled, 100 pM, 200 pM, 300 pM and 400 pM) of nanostars. All three sets of box plots show that the spatial periods tend to increase with increasing nanostar concentrations. b) We used a single mean spatial period value on the Y-axis to represent the entire sample for each group. For this purpose, we calculated the mean value from the individual box plots in (a) and applied a first-order polynomial fit. The $R^{2}$ and r values are the coefficient of determination and Pearson correlation, respectively.

The results from experimental group 3 are presented in Fig. 4(a). Figure 4(a) shows three sets of box plots, each of them from different individual biological donors. All the box plots in Fig. 4(a) are formed using mean spatial periods from en face images similar to Fig. 3. From each donor, we imaged five samples with different concentrations of nanostars, including an unlabelled control. The box plots from all three donors indicate a gradual increase in the mean spatial period values with respect to the increasing concentrations of nanostars. The results in Fig. 4(a) are in agreement with the results in Fig. 3. The mean spatial periods tend to increase with higher concentrations of nanostars. This trend is reproduced across experimental groups 1 and 2, and 3. To analyse the trend further, we plotted the mean value from each boxplot in 4(a) and applied a first-order polynomial fit. The results are shown in 4(b). The $R^{2}$ and r values indicated on each plot represent the coefficient of determination and Pearson correlation, respectively. High $R^{2}$ and r values indicate a strong linear relationship between the nanostar concentration and spatial period.

**Fig. 5.**
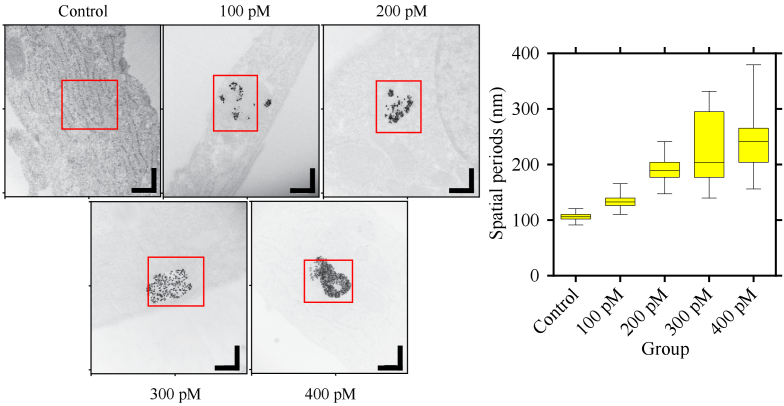
TEM images from samples labelled with different concentrations of nanostars. We performed Fourier analysis on the line profiles from the areas selected by the red rectangle. The Fourier analysis returned the energy contribution from available spatial periods. The box plot was constructed using the median values of spatial period profiles. We can observe from the box plot that the spatial periods increase with the increasing concentrations of nanostars. The result in the box plot is in agreement with the result from nsOCT analysis on experimental group 3. The scale bar on the images represents 400 nm.

To validate the nsOCT results from experimental group 3, we acquired high-resolution images using a transmission electron microscope from one of the three donors. The spatial periods were calculated from regions where the nanostar intake was observed. To analyse the spatial periods, we performed FFT on approximately 200 line profiles across the regions marked by the red-coloured rectangles and obtained the energy contributions from different spatial periods. Then the median value was calculated from the FFT of each line profile. The median values from all line profiles were combined to form the box plots in Fig. 5. The TEM images contain 928×1024 pixels, and the scale bars on the images represent 400 nm. The Fourier analysis on the TEM images also confirms that the spatial periods tend to increase with the increasing concentrations of nanostars.

**Fig. 6.**
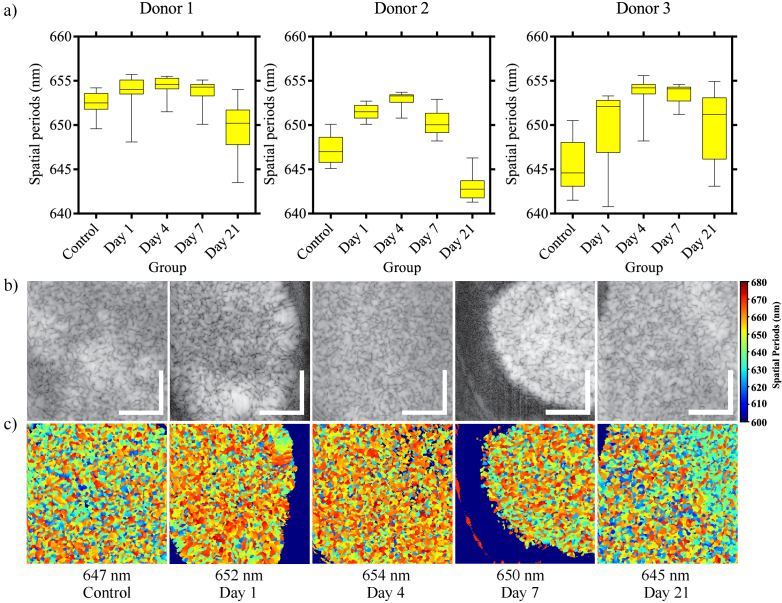
The potential of the nsOCT technique to detect the morphological changes associated with chondrogenic differentiation in MSC pellets. a) shows the mean spatial periods from different time points of chondrogenic differentiation and three different donors. We can see from the box plots that the spatial periods tend to increase until day 4 and decreases after that. b) shows the conventional intensity-based representative OCT images from donor 2. c) shows the nsOCT images corresponding to the conventional intensity-based images from donor 2 on 3(b). The mean spatial periods calculated from the nsOCT images are mentioned below the respective nsOCT images. The general trend from all three donors reveals that the spatial period tends to increase up to day 4 and decreases after that. The scale bar represents 0.1mm .

**Fig. 7.**
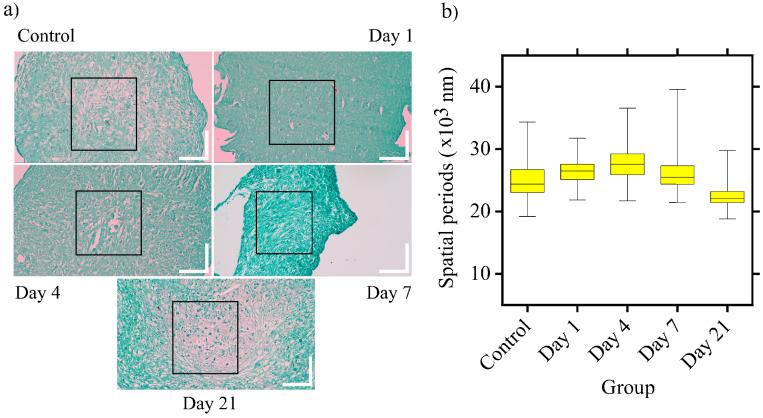
a) Histology images from the samples at different stages of chondrogenic differentiation. The scale bar represents 100 µm. b) We performed Fourier analysis on the line profiles within the rectangular areas selected. After the Fourier transform, we calculated the median value from the spatial period versus energy contribution for each line profile. These median spatial periods from all line profiles are used in the respective boxplots.

To demonstrate the potential of nsOCT to detect structural changes associated with chondrogenic differentiation in MSC pellets, we analysed different stages of chondrogenic differentiation in cells from three different donors (experimental group 4). To mimic the natural hypoxic 3-D environment of cartilage, MSCs were exposed to (TGF)−β3 in micromass culture under low oxygen conditions. From each donor, MSC pellets were maintained under these conditions for either one, four, seven or twenty-one days (see section. 2.6). We also retained an undifferentiated negative control in pellet culture for 21 days. To form the box plots in Fig. 6(a), we followed steps similar to those in Fig. 4 and 3. We acquired 3-dimensional OCT images from each sample with 300 pixels in each lateral direction. From these 3-D volumes, we formed en face images at every slice along the depth. We calculated the mean spatial period corresponding to that pixel/depth from these en face images. These mean spatial period values from all the depth pixels are represented in the box plots. The box plots in Fig. 6(a) show that the mean spatial periods tend to increase up to four days. After that, the spatial periods tend to decrease. This trend is consistent among all three donors. Chondrogenic differentiation is the process by which cartilage is formed from condensed mesenchyme tissue [58]. The process of stem cell chondrogenic differentiation occurs in two separate phases: mesenchymal condensation and subsequent differentiation into chondrocytes [59]. As the cells progress through different stages of chondrogenic differentiation, changes occur in the structure and organization of cells and the extracellular matrix. The early stages of the process involve forming a fibronectin-rich precartilage condensation matrix. This precartilage condensation matrix is then transformed into an aggrecan-rich cartilage matrix [60]. We hypothesize that the increase in spatial periods or the size of dominant structure up to day 4 in the nsOCT analysis indicates the formation of a precartilage condensation matrix. The spatial period tends to decrease after day 4. This decrease in the spatial period could be because the precartilage matrix transforms into an aggrecan-rich cartilage matrix by this stage. Figures 6(a) and 6(b) show the representative conventional OCT and nsOCT enface images from one of the donors at different time points. To confirm the nsOCT results, we generated histology images from one of the donors at all the time points (Fig. 7(a)). The details regarding the preparation of samples for histology imaging are presented in section 2.7. We then performed Fourier analysis on the regions marked by the black rectangles on the histology images. The histology images contain 3264 x 1836 pixels, and the scale bars represent 100 µm. Unfortunately, the resolution of the histology images is not sufficient to compute the spatial periods at the nano-scale, as was done using nsOCT. The region marked by the black rectangle was arbitrarily selected in a location central to the histology image. Similar to the analysis performed in Fig. 5, FFT was performed on approximately 1000 line profiles across this region to obtain the energy contributions from available spatial periods. From this data, we calculated the median spatial period. Thus, each intensity profile along the selected line has a median spatial period associated with it. The median spatial periods from all the line profiles within the selected region were used to create the boxplot in Fig. 7(b). It can be observed that the variations in the spatial period follow the same pattern as that of nsOCT images. Even though the spatial periods detected from histology images are in the micrometre range, the trend in spatial period changes is similar to those observed in the nsOCT analysis. The spatial periods increase until day 4 and begin to decrease after that. Therefore, the results from the Fourier analysis on histology images correlate with the results obtained from the nsOCT analysis. From the results explained above, we can conclude that the nsOCT is sensitive to the structural changes in MSC pellets associated with different stages of chondrogenic differentiation.

## 4. Conclusion

The experimental results show that the nsOCT technique can distinguish MSC pellets labelled with varying concentrations of gold plasmonic nanostars. We observed that the spatial periods tend to increase with the increasing concentrations of nanostars. While several imaging modalities use nanostars as a contrast agent, nsOCT can give information directly related to the structural changes associated with the labelling. The nanoscale sensitivity permits the detection of nanostar concentration changes as small as 100 pM. We supported the findings from the nsOCT technique using corresponding high-resolution TEM images. We also improved the MSC pellets chondrogenesis differentiation model by providing a more complete analysis of the trend in structural changes that occur during cartilage development over time. The results demonstrated that nsOCT could detect nanoscale structural changes in different stages of the chondrogenic differentiation of stem cells, which are not detectable using conventional OCT. The spatial period increased during the progression in the formation of the precartilage condensation matrix and decreased during its transformation into an aggrecan-rich cartilage matrix. We confirmed the spatial period changes associated with chondrogenic differentiation obtained from nsOCT analysis by performing a Fourier analysis on histology images from corresponding samples. To summarise; the attributes of nsOCT such as real-time imaging, high spatial resolution, high temporal resolution, nanoscale sensitivity to structural alterations and ability to image for longer durations make it an ideal candidate to assist in stem cell therapy. While nsOCT does not require any labelling, it is sensitive to contrast agents such as nanostars. This nanoscale sensitivity enables the detection of the stem cells labelled with different contrast agents. The nsOCT can be used as a complementary imaging technique along with other imaging modalities to successfully contribute toward novel research opportunities such as determining the location and quantity of the cells, visualizing the in vivo distribution of stem cells, tracking the migration to the targeted cells and determining their long-term fate. The major limitation of the technique is that the detected structural or morphological changes are non-specific compared to non-linear imaging techniques that use labels, like photoacoustic imaging or fluorescence microscopy. Nanosensitive OCT technique can be implemented in conjunction with other imaging modalities to gain some insights into the mechanisms underpinning the morphological changes.

## Data Availability

Data underlying the results presented in this paper are available upon request.

## References

[1] Friedenstein A. J. Chailakhjan R. K. Lalykina K. S., “The development of fibroblast colonies in monolayer cultures of guinea-pig bone marrow and spleen cells,” Cell Proliferation 3(4), 393–403 (1970). 10.1111/j.1365-2184.1970.tb00347.x 5523063

[2] Barry F. Murphy M., “Mesenchymal stem cells in joint disease and repair,” Nat. Rev. Rheumatol. 9(10), 584–594 (2013). 10.1038/nrrheum.2013.109 23881068

[3] Bunnell B. A. Estes B. T. Guilak F. Gimble J. M., “Differentiation of adipose stem cells,” Methods Mol. Biol. (Clifton, N.J.) 456, 155–171 (2008). 10.1007/978-1-59745-245-8_12 18516560

[4] Troyer D. L. Weiss M. L., “Concise review: Wwatharton’s jelly-derived cells are a primitive stromal cell population,” Stem Cells 26(3), 591–599 (2008). 10.1634/stemcells.2007-0439 18065397 PMC3311226

[5] Weiss M. L. Troyer D. L., “Stem cells in the umbilical cord,” Stem Cell Rev. 2(2), 155–162 (2006). 10.1007/s12015-006-0022-y 17237554 PMC3753204

[6] Tögel F. Hu Z. Weiss K. Isaac J. Lange C. Westenfelder C., “Administered mesenchymal stem cells protect against ischemic acute renal failure through differentiation-independent mechanisms,” Am. J. Physiol. Ren. Physiol. 289(1), F31–F42 (2005). 10.1152/ajprenal.00007.2005 15713913

[7] Maitra B. Szekely E. Gjini K. Laughlin M. J. Dennis J. Haynesworth S. E. Koç O. N., “Human mesenchymal stem cells support unrelated donor hematopoietic stem cells and suppress T-cell activation,” Bone Marrow Transplant. 33(6), 597–604 (2004). 10.1038/sj.bmt.1704400 14716336

[8] Tse W. T. Pendleton J. D. Beyer W. M. Egalka M. C. Guinan E. C., “Suppression of allogeneic T-cell proliferation by human marrow stromal cells: Implications in transplantation,” Transplantation 75(3), 389–397 (2003). 10.1097/01.TP.0000045055.63901.A9 12589164

[9] Bartholomew A. Sturgeon C. Siatskas M. Ferrer K. McIntosh K. Patil S. Hardy W. Devine S. Ucker D. Deans R. Moseley A. Hoffman R., “Mesenchymal stem cells suppress lymphocyte proliferation in vitro and prolong skin graft survival in vivo,” Exp. Hematol. 30(1), 42–48 (2002). 10.1016/S0301-472X(01)00769-X 11823036

[10] Kassem M. Kristiansen M. Abdallah B. M., “Mesenchymal stem cells: cell biology and potential use in therapy,” Basic Clin. Pharmacol. Toxicol. 95, 209–214 (2004). 10.1111/j.1742-7843.2004.pto950502.x 15546474

[11] Ripa R. S. Haack-Sørensen M. Wang Y. Jørgensen E. Mortensen S. Bindslev L. Friis T. Kastrup J., “Bone marrow derived mesenchymal cell mobilization by granulocyte-colony stimulating factor after acute myocardial infarction: Results from the Stem Cells in Myocardial Infarction (STEMMI) trial,” Circulation 116, I24–30 (2007). 10.1161/CIRCULATIONAHA.106.678649 17846310

[12] Chen S.-L. Fang W.-W. Ye F. Liu Y.-H. Qian J. Shan S.-J. Zhang J.-J. Chunhua R. Z. Liao L.-M. Lin S. Sun J.-P., “Effect on left ventricular function of intracoronary transplantation of autologous bone marrow mesenchymal stem cell in patients with acute myocardial infarction,” Am. J. Cardiol. 94(1), 92–95 (2004). 10.1016/j.amjcard.2004.03.034 15219514

[13] Leahy M. Thompson K. Zafar H. Alexandrov S. Foley M. O’Flatharta C. Dockery P., “Functional imaging for regenerative medicine,” Stem Cell Res. Ther. 7(1), 57 (2016). 10.1186/s13287-016-0315-2 27095443 PMC4837501

[14] James S. Neuhaus K. Murphy M. Leahy M., “Contrast agents for photoacoustic imaging: a review of stem cell tracking,” Stem Cell Res. Ther. 12(1), 511 (2021). 10.1186/s13287-021-02576-3 34563237 PMC8467005

[15] Zhou R. Acton P. D. Ferrari V. A., “Imaging Stem Cells Implanted in Infarcted Myocardium,” J. Am. Coll. Cardiol. 48(10), 2094–2106 (2006). 10.1016/j.jacc.2006.08.026 17112999 PMC2597078

[16] Zhang S. J. Wu J. C., “Comparison of Imaging Techniques for Tracking Cardiac Stem Cell Therapy,” J. Nucl. Medicine 48(12), 1916–1919 (2007). 10.2967/jnumed.107.043299 PMC3638042 18056330

[17] Roeder E. Henrionnet C. Goebel J. C. Gambier N. Beuf O. Grenier D. Chen B. Vuissoz P.-A. Gillet P. Pinzano A., “Dose-Response of Superparamagnetic Iron Oxide Labeling on Mesenchymal Stem Cells Chondrogenic Differentiation: A Multi-Scale In Vitro Study,” PLoS One 9(5), e98451 (2014). 10.1371/journal.pone.0098451 24878844 PMC4039474

[18] Meir R. Betzer O. Motiei M. Kronfeld N. Brodie C. Popovtzer R., “Design principles for noninvasive, longitudinal and quantitative cell tracking with nanoparticle-based CT imaging,” Nanomedicine 13(2), 421–429 (2017). 10.1016/j.nano.2016.09.013 27720990

[19] Chhour P. Naha P. C. O’Neill S. M. Litt H. I. Reilly M. P. Ferrari V. A. Cormode D. P., “Labeling monocytes with gold nanoparticles to track their recruitment in atherosclerosis with computed tomography,” Biomaterials 87, 93–103 (2016). 10.1016/j.biomaterials.2016.02.009 26914700 PMC4783300

[20] Schroeder T., “Imaging stem-cell-driven regeneration in mammals,” Nature 453(7193), 345–351 (2008). 10.1038/nature07043 18480816

[21] Ntziachristos V., “Going deeper than microscopy: The optical imaging frontier in biology,” Nat. Methods 7(8), 603–614 (2010). 10.1038/nmeth.1483 20676081

[22] Wang L. V., “Multiscale photoacoustic microscopy and computed tomography,” Nat. Photonics 3(9), 503–509 (2009). 10.1038/nphoton.2009.157 20161535 PMC2802217

[23] Zackrisson S. Y. van de Ven S. M. W. Gambhir S. S., “Light in and sound out: Emerging translational strategies for photoacoustic imaging,” Cancer Res. 74(4), 979–1004 (2014). 10.1158/0008-5472.CAN-13-2387 24514041 PMC3944207

[24] Erpelding T. N. Kim C. Pramanik M. Jankovic L. Maslov K. Guo Z. Margenthaler J. A. Pashley M. D. Wang L. V., “Sentinel Lymph Nodes in the Rat: Noninvasive Photoacoustic and US Imaging with a Clinical US System1,” Radiology 256(1), 102–110 (2010). 10.1148/radiol.10091772 20574088 PMC2897692

[25] Raghavan V. O’Flatharta C. Dwyer R. Breathnach A. Zafar H. Dockery P. Wheatley A. Keogh I. Leahy M. Olivo M., “Dual plasmonic gold nanostars for photoacoustic imaging and photothermal therapy,” Nanomedicine 12(5), 457–471 (2017). 10.2217/nnm-2016-0318 28181456

[26] Huang D. Swanson E. A. Lin C. P. Schuman J. S. Stinson W. G. Chang W. Hee M. R. Flotte T. Gregory K. Puliafito C. A. Et A., “Optical coherence tomography,” Science 254(5035), 1178–1181 (1991). 10.1126/science.1957169 1957169 PMC4638169

[27] Apelian C. Harms F. Thouvenin O. Boccara A. C., “Dynamic full field optical coherence tomography: Subcellular metabolic contrast revealed in tissues by interferometric signals temporal analysis,” Biomed. Opt. Express 7(4), 1511–1524 (2016). 10.1364/BOE.7.001511 27446672 PMC4929658

[28] Tsai C.-Y. Shih C.-H. Chu H.-S. Hsieh Y.-T. Huang S.-L. Chen W.-L., “Submicron spatial resolution optical coherence tomography for visualising the 3D structures of cells cultivated in complex culture systems,” Sci. Rep. 11(1), 3492 (2021). 10.1038/s41598-021-82178-4 33568705 PMC7875968

[29] Liu L. Gardecki J. A. Nadkarni S. K. Toussaint J. D. Yagi Y. Bouma B. E. Tearney G. J., “Imaging the subcellular structure of human coronary atherosclerosis using micro–optical coherence tomography,” Nat. Med. 17(8), 1010–1014 (2011). 10.1038/nm.2409 21743452 PMC3151347

[30] Wang R. K. Nuttall A. L., “Phase-sensitive optical coherence tomography imaging of the tissue motion within the organ of Corti at a subnanometer scale: A preliminary study,” J. Biomed. Opt. 15(5), 056005 (2010). 10.1117/1.3486543 21054099 PMC2948044

[31] Hillman T. R. Adie S. G. Seemann V. Armstrong J. J. Jacques S. L. Sampson D. D., “Correlation of static speckle with sample properties in optical coherence tomography,” Opt. Lett. 31(2), 190–192 (2006). 10.1364/OL.31.000190 16441026

[32] Yi J. Radosevich A. J. Rogers J. D. Norris S. C. P. Çapoğlu İ. R. Taflove A. Backman V., “Can OCT be sensitive to nanoscale structural alterations in biological tissue?” Opt. Express 21(7), 9043–9059 (2013). 10.1364/OE.21.009043 23571994 PMC3641881

[33] Alexandrov S. A. Subhash H. M. Zam A. Leahy M., “Nano-sensitive optical coherence tomography,” Nanoscale 6(7), 3545–3549 (2014). 10.1039/C3NR06132A 24595392

[34] Alexandrov S. Subhash H. Leahy M., “Nanosensitive optical coherence tomography for the study of changes in static and dynamic structures,” Quantum Electron. 44(7), 657–663 (2014). 10.1070/QE2014v044n07ABEH015487

[35] Alexandrov S. McNamara P. M. Das N. Zhou Y. Lynch G. Hogan J. Leahy M., “Spatial frequency domain correlation mapping optical coherence tomography for nanoscale structural characterization,” Appl. Phys. Lett. 115(12), 121105 (2019). 10.1063/1.5110459

[36] Das N. Sergey A. Zhou Y. Gilligan K. E. Dwyer R. M. Leahy M., “Nanoscale structure detection and monitoring of tumour growth with optical coherence tomography,” Nanoscale Adv. 2(7), 2853–2858 (2020). 10.1039/D0NA00371A

[37] Zhou Y. Alexandrov S. Nolan A. Das N. Dey R. Leahy M., “Noninvasive detection of nanoscale structural changes in cornea associated with cross-linking treatment,” J. Biophotonics 13(6), e201960234 (2020). 10.1002/jbio.201960234 32067338

[38] Das N. Alexandrov S. Gilligan K. E. Dwyer R. M. Saager R. B. Ghosh N. Leahy M., “Characterization of nanosensitive multifractality in submicron scale tissue morphology and its alteration in tumor progression,” J. Biomed. Opt. 26(1), 016003 (2021). 10.1117/1.JBO.26.1.016003 33432788 PMC7797786

[39] Alexandrov S. Arangath A. Zhou Y. Murphy M. Duffy N. Neuhaus K. Shaw G. McAuley R. Leahy M., “Accessing depth-resolved high spatial frequency content from the optical coherence tomography signal,” Sci. Rep. 11(1), 17123 (2021). 10.1038/s41598-021-96619-7 34429483 PMC8385072

[40] Dsouza R. Won J. Monroy G. L. Hill M. C. Porter R. G. Novak M. A. Boppart S. A., “In vivo detection of nanometer-scale structural changes of the human tympanic membrane in otitis media,” Sci. Rep. 8(1), 8777 (2018). 10.1038/s41598-018-26514-1 29884809 PMC5993811

[41] Lal C. Alexandrov S. Rani S. Zhou Y. Ritter T. Leahy M., “Nanosensitive optical coherence tomography to assess wound healing within the cornea,” Biomed. Opt. Express 11(7), 3407–3422 (2020). 10.1364/BOE.389342 33014541 PMC7510923

[42] Buckwalter J. A. Mankin H. J., “Articular cartilage: Degeneration and osteoarthritis, repair, regeneration, and transplantation,” Instructional Course Lectures 47, 487–504 (1998). 9571450

[43] Iannone F. Lapadula G., “The pathophysiology of osteoarthritis,” Aging: Clin. Exp. Res. 15(5), 364–372 (2003). 10.1007/BF03327357 14703002

[44] Akkiraju H. Nohe A., “Role of chondrocytes in cartilage formation, progression of osteoarthritis and cartilage regeneration,” J. Dev. Biol. 3(4), 177–192 (2015). 10.3390/jdb3040177 27347486 PMC4916494

[45] Loeser R. F., “Age-related changes in the musculoskeletal system and the development of osteoarthritis,” Clin. Geriatr. Med. 26(3), 371–386 (2010). 10.1016/j.cger.2010.03.002 20699160 PMC2920876

[46] Khoury C. G. Vo-Dinh T., “Gold nanostars for surface-enhanced Raman scattering: synthesis, characterization and optimization,” J. Phys. Chem. C 112(48), 18849–18859 (2008). 10.1021/jp8054747 PMC3748989 23977403

[47] Xia Y. Gilroy K. D. Peng H.-C. Xia X., “Seed-mediated growth of colloidal metal nanocrystals,” Angew. Chem. Int. Ed. 56(1), 60–95 (2017). 10.1002/anie.201604731 27966807

[48] Scarabelli L., “Recent advances in the rational synthesis and self-assembly of anisotropic plasmonic nanoparticles,” Pure Appl. Chem. 90(9), 1393–1407 (2018). 10.1515/pac-2018-0510

[49] Murphy J. M. Fink D. J. Hunziker E. B. Barry F. P., “Stem cell therapy in a caprine model of osteoarthritis,” Arthritis Rheum. 48, 3464–3474 (2003). 10.1002/art.11365 14673997

[50] Dominici M. Le Blanc K. Mueller I. Slaper-Cortenbach I. Marini F. Krause D. Deans R. Keating A. Prockop D. Horwitz E., “Minimal criteria for defining multipotent mesenchymal stromal cells. The International Society for Cellular Therapy position statement,” Cytotherapy 8(4), 315–317 (2006). 10.1080/14653240600855905 16923606

[51] Murphy J. M. Dixon K. Beck S. Fabian D. Feldman A. Barry F., “Reduced chondrogenic and adipogenic activity of mesenchymal stem cells from patients with advanced osteoarthritis,” Arthritis Rheum. 46, 704–713 (2002). 10.1002/art.10118 11920406

[52] Kavalkovich K. W. Boynton R. E. Mary Murphy J. Barry F., “Chondrogenic differentiation of human mesenchymal stem cells within an alginate layer culture system,” In Vitro Cell Dev. Biol. Anim. 38(8), 457–466 (2002). 10.1290/1071-2690(2002)038<0457:CDOHMS>2.0.CO;2 12605540

[53] Barry F. Boynton R. E. Liu B. Murphy J. M., “Chondrogenic Differentiation of Mesenchymal Stem Cells from Bone Marrow: Differentiation-Dependent Gene Expression of Matrix Components,” Exp. Cell Res. 268(2), 189–200 (2001). 10.1006/excr.2001.5278 11478845

[54] Kamaleddin M. A., “Nano-ophthalmology: Applications and considerations,” Nanomedicine 13(4), 1459–1472 (2017). 10.1016/j.nano.2017.02.007 28232288

[55] Kavalaraki A. Spyratou E. Kouri M. A. Efstathopoulos E. P., “Gold nanoparticles as contrast agents in ophthalmic imaging,” Optics 4(1), 74–99 (2023). 10.3390/opt4010007

[56] Nguyen V.-P. Li Y. Henry J. Zhang W. Aaberg M. Jones S. Qian T. Wang X. Paulus Y. M., “Plasmonic gold nanostar-enhanced multimodal photoacoustic microscopy and optical coherence tomography molecular imaging to evaluate choroidal neovascularization,” ACS Sens. 5(10), 3070–3081 (2020). 10.1021/acssensors.0c00908 32921042 PMC8121042

[57] Park J. Ha M. K. Yang N. Yoon T. H., “Flow cytometry-based quantification of cellular au nanoparticles,” Anal. Chem. 89(4), 2449–2456 (2017). 10.1021/acs.analchem.6b04418 28192941

[58] Marín-Llera J. C. Garciadiego-Cázares D. Chimal-Monroy J., “Understanding the cellular and molecular mechanisms that control early cell fate decisions during appendicular skeletogenesis,” Front. Genet. 10, 1 (2019). 10.3389/fgene.2019.00977 31681419 PMC6797607

[59] Mackay A. M. Beck S. C. Murphy J. M. Barry F. P. Chichester C. O. Pittenger M. F., “Chondrogenic Differentiation of Cultured Human Mesenchymal Stem Cells from Marrow,” Tissue Eng. 4(4), 415–428 (1998). 10.1089/ten.1998.4.415 9916173

[60] Singh P. Schwarzbauer J. E., “Fibronectin and stem cell differentiation - lessons from chondrogenesis,” J. Cell Sci. 125(16), 3703–3712 (2012). 10.1242/jcs.095786 22976308 PMC3462078

